# Endoscopic endonasal resection via a transsphenoidal and transpterygoid approach for sphenoid ridge meningioma extending into the sphenoid sinus: A case report and literature review

**DOI:** 10.1016/j.ijscr.2019.06.003

**Published:** 2019-06-12

**Authors:** Hidenori Yokoi, Satoru Kodama, Keisuke Maruyama, Masachika Fujiwara, Yoshiaki Shiokawa, Koichiro Saito

**Affiliations:** a Department of Otolaryngology, Head and Neck Surgery, Kyorin University School of Medicine, Tokyo, Japan; b Kodama Ear, Nose, and Throat Clinic, Oita, Japan; c Department of Neurosurgery, Kyorin University School of Medicine, Tokyo, Japan; d Department of Pathology, Kyorin University School of Medicine, Tokyo, Japan

**Keywords:** EEA, endoscopic endonasal approach, Sphenoidal ridge meningioma, Staged approach

## Abstract

**Introduction:**

Large sphenoid ridge meningiomas are surgically challenging because of their extension to adjacent structures. Extracranial meningiomas are very rare; most are of the secondary type and have an intracranial origin. Improved surgical methods are required for treatment of this entity.

**Presentation of case:**

Here, we describe a case of a huge sphenoid ridge meningioma extending into the sphenoid sinus that was resected by staged transcranial and endoscopic endonasal resection via a transsphenoidal and transpterygoid approach. It was possible to excise the tumor from the sphenoid sinus in a minimally invasive manner via this approach.

**Discussion and conclusion:**

The transsphenoidal and transpterygoid approach was useful for providing a surgical field that allowed adequate visualization for removal of a sphenoid tumor that had expanded well into the lateral fossa of the sphenoid bone.

## Highlights

• Extracranial meningiomas are rare, of a secondary type, and of intracranial origin.
• We describe a case of sphenoid ridge meningioma extending into the sphenoid sinus.
• The tumor was resected by staged transcranial and endoscopic endonasal resection.
• A transsphenoidal and transpterygoid approach was used.
• This approach provided adequate visualization for removal of the tumor.

## 1 Introduction

Meningiomas are benign tumors that account for 13%–26% of all primary intracranial tumors [1]. Extracranial meningiomas are very rare, and are reported to account for 1%–2% of all meningiomas [2,3]. Depending on the anatomical location of these lesions, extracranial meningiomas are clinically classified as primary or secondary [4]. Most extracranial meningiomas are secondary extensions from the intracranial lesion [5,6].

The only curative treatment for meningioma is complete surgical extirpation. However, because of the tendency of this tumor to extend into adjacent structures, such as the cavernous sinus, the anterior clinoid process, the middle fossa, and greater and lesser sphenoid wings, resection of these tumors is challenging [7].

Technological advances in recent years mean that surgical procedures for various pathological conditions, including tumors situated in the paranasal sinuses or the skull base, can be performed via a minimally invasive endoscopic endonasal approach (EEA).　

Here we report the case of a patient in whom a sphenoid ridge meningioma in the sphenoid sinus was excised by staged surgery that consisted of a craniotomy for the intracranial portion and an endoscopic endonasal resection via a transsphenoidal and transpterygoid approach for the extracranial portion in the sphenoid sinus. The work has been reported in line with the SCARE criteria [8].

## 2 Presentation of case

**Fig. 1:**
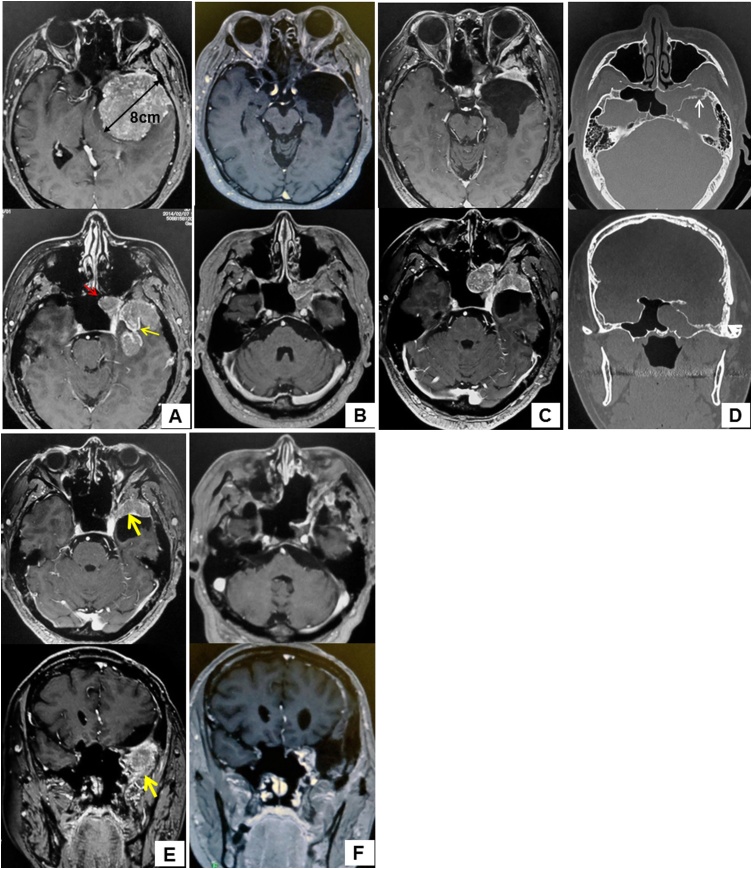
**(A)** A magnetic resonance image with gadolinium enhancement obtained at the initial visit shows an ovoid tumor with a maximum diameter of 8 cm on the left sphenoid ridge. The tumor was exerting strong pressure on the brain and was progressing further from the tip of the temporal bone (yellow arrow) into the sphenoid sinus (red arrow). **(B)** A magnetic resonance image with gadolinium enhancement obtained after a subtotal tumor excision by craniotomy. The temporal lobe tumor was removed but the tumor at the tip of the temporal bone to the sphenoid sinus remained. **(C)** A magnetic resonance image with gadolinium enhancement obtained 20 months after the subtotal tumor excision. The tumor remaining at the tip of the temporal bone to the sphenoid sinus was increasing in size. **(D)** A preoperative non-contrast-enhanced computed tomography image obtained before surgery via the endoscopic endonasal approach shows that the lateral recess of the sphenoid sinus was markedly enlarged and there was a soft tissue shadow occupying the left sphenoid sinus. Erosion was confirmed in the part of the bone bordering the lateral tip of the temporal lobe, suggesting a bone fracture in the order of several millimeters at this site (white arrow). **(E)** A magnetic resonance image with gadolinium enhancement obtained after surgery via the endoscopic endonasal approach confirming that the tumor within the sphenoid sinus had been completely removed, with residual tumor tissue remaining only at the tip of the left temporal lobe (yellow arrow). **(F)** A magnetic resonance image with gadolinium enhancement obtained after total resection of the residual tumor via craniotomy. No presence or recurrence of the tumor within the cranium or sphenoid sinus was observed.

A 54-year-old man presented with a grade 4/5 right-sided hemiplegia. Neuroimaging studies revealed a left-sided sphenoid ridge meningioma; the tumor was exerting pressure on the brain parenchyma and extended further from the temporal lobe into the sphenoid sinus (Fig. 1A). The patient also had severe diabetes mellitus.

Surgical resection using a staged transcranial and EEA approach was planned to minimize the risk of surgical complications in view of the patient’s preoperative hemiplegia, the size of the tumor, and its extension into the sphenoid sinus, as well as the patient’s generally compromised state of health. Cerebral angiography revealed that the tumor was hypervascular, being supplied by branches of the left middle meningeal artery. Therefore, embolization using *N*-butyl cyanoacrylate was performed on the day before the first surgical resection. Subtotal tumor excision via craniotomy was performed by the neurosurgeons, but the portion of the tumor extending anterior to the tip of the temporal bone to the sphenoid sinus was not removed (Fig. 1B). The pathological diagnosis was a World Health Organization grade I transitional meningioma [1] (Fig. 3A). The MIB-1 index was less than 1% (Fig. 3D). The patient’s motor weakness resolved completely after 6 months of postoperative rehabilitation. After 2 years of regular postoperative follow-up, the residual tumor was noted to have increased in size (Fig. 1C). Therefore, the otolaryngologists planned to remove the tumor in the sphenoid sinus via EEA. Computed tomography revealed that the tumor had grown considerably in the lateral recess of the sphenoid sinus and that there was a soft tissue shadow occupying the left sphenoid sinus (Fig. 1D). Erosion in the order of several millimeters was confirmed in the portion of the bone bordering the lateral tip of the temporal lobe. We suspected that the intracranial tumor had extended into the sphenoid sinus through the small area of eroded bone.

**Fig. 2:**
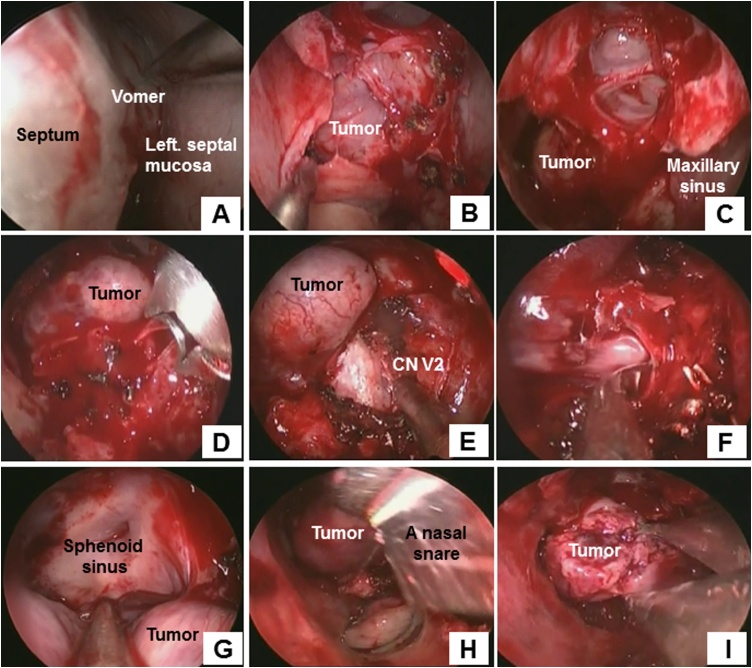
**(A)** Reconstruction of the nasal septum. The left septal mucosa was detached under the periosteum up to the vomer. **(B, C)** After the vomer and mucosa of the anterior wall of the sphenoid sinus were removed, part of the tumor was clearly visualized. **(D)** The left paranasal cavity was opened and a modified endoscopic medial maxillectomy was performed. **(E)** The sphenopalatine and descending pharyngeal arteries were clipped and severed. The communicating branch of the nerve was severed at this time. **(F)** Confirmation of V2 on bone of the posterior wall of the pterygopalatine fossa. **(G, H)** The left lateral recess of the sphenoid sinus was approached. Examination of the overall image of the tumor revealed that the base was in the lateral wall of the left sphenoid sinus. **(I)** The base of the tumor was subsequently severed using a nasal snare. (J, **K)** The body of the tumor was removed. **(L)** After removing the remaining tumor at the base using forceps, packing was performed and the surgery was completed.

**Fig. 3:**
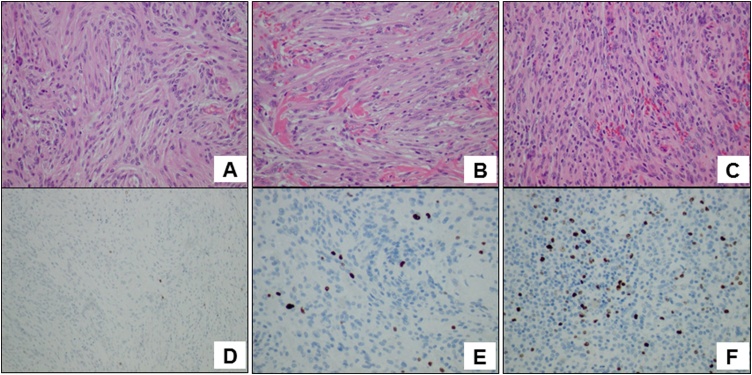
**(A)** Hematoxylin-eosin staining at the initial craniotomy. The relatively uniform-type circular nucleus and spindle cells with abundant cytoplasm exhibited substantial hyperplasia. This was diagnosed as transitional (mixed) meningioma, World Health Organization grade I. **(B)** Hematoxylin-eosin staining at the time of the endoscopic endonasal approach. **(C)** Hematoxylin-eosin staining at the time of the second craniotomy showing nuclear enlargement and increased nuclear density. **(D)** Anti-Ki-67 antibody staining at the initial craniotomy. The MIB-1 index was 1% or less. **(E)** Anti-Ki-67 antibody staining at the time of the endoscopic endonasal approach. The MIB-1 index was approximately 5%. **(F)** Anti-Ki-67 antibody staining at the time of the second craniotomy. The MIB-1 index was approximately 15%. However, mitosis, among other processes, was limited, and there were no malignant findings.

An EEA was performed to excise the tumor in the sphenoid sinus. First, the nasal septum was partially resected via the left nasal cavity to allow better visualization of the posterior nasal cavity. The septal mucosa was detached under the periosteum up to the vomer (Fig. 2A). After the vomer was removed using a drill, the mucosa of the anterior wall of the sphenoid sinus was removed, affording a clear view of one portion of the tumor (Fig. 2B). The lower half of the left middle nasal turbinate and the superior nasal turbinate were then excised to afford clear and complete visualization of the sphenoid sinus. Next, the left paranasal cavity was opened and a modified endoscopic medial maxillectomy (EM3) [9] was performed (Fig. 2C). After the posterior wall of the left maxillary sinus was skeletonized with a drill, the sphenopalatine and descending pharyngeal arteries were clipped and cut (Fig. 2D). At this time, the communicating branch of the nerve was also severed. After confirmation of the maxillary nerve (CN V2) at the bone on the posterior wall of the pterygopalatine fossa (Fig. 2E), the left lateral recess of the sphenoid sinus was approached (Fig. 2F, G). The base of the tumor was in the lateral wall of the left sphenoid sinus and penetrated from the area just anterior to the tip of the temporal bone to the sphenoid sinus via a pin-sized area of bone erosion, so the tumor was removed using a nasal snare (Fig. 2H). The body of the tumor was then removed (Fig. 2I). Next, the remaining portion of the tumor was removed at the base using forceps. Packing was then placed and the surgery was completed. The MIB-1 index of the sphenoidal portion was 5% with no difference in the pathology (Fig. 3B, E). Six months later, the neurosurgeons performed a craniotomy to remove the tumor from the area extending from just anterior to the tip of the temporal bone to the sphenoid sinus (Fig. 1E, F). Thereafter, the MIB-1 index increased to 15% with nuclear enlargement and increased nuclear density (Fig. 3C, F). The patient’s postoperative course was favorable and there has been no evidence of tumor recurrence in the 36 months since the last surgery.

## 3 Discussion

Meningiomas are essentially benign tumors that originate in the dura mater and rarely extend outside of the cranium. However, there are some reports of secondary extension of intracranial meningiomas into sites such as the orbit, middle ear, nasal cavity, nasopharynx, and paranasal sinuses [3,10]. This type of tumor may enter the orbit through a preformed bony pathway, a surgical defect, or one of the foramina in the skull [3]. The tumor may also enter the nasal cavity or nasopharynx through the cribriform plate or via the paranasal sinuses and infratemporal-parotid region through the floor of the middle cranial fossa [3].

Meningiomas often compress the surrounding tissue without infiltrating it [11]; however, they can extend into the extracranial tissue, resulting in a skull bone reaction to the tumor in 30%–60% of patients [12]. Our patient had a very large sphenoid ridge meningioma that extended into the sphenoid sinus. It was suspected that the intracranial tumor had penetrated throughout a small hole in an area of the eroded bone in the lateral recess of the sphenoid sinus, but there was no obvious tumor infiltration into the surrounding tissue.

Large intracranial meningiomas usually cause symptoms associated with neurological deficits. Our patient presented with grade 4/5 right-sided hemiplegia, so we selected staged surgery to avoid the complications that can occur after aggressive surgery as much as possible. The residual tumor in the sphenoid sinus increased in size in the 2 years following craniotomy. Therefore, we planned surgery for the sphenoidal lesion via an EEA.

Use of a combined transcranial and EEA approach to remove a spheno-orbital meningioma extending into the sphenoid sinus, pterygopalatine fossa, and infratemporal fossa has been reported previously [13]. This combined strategy was used to remove the tumor and considered to be a safe and feasible surgical option for spheno-orbital meningioma with a large skull base defect penetrating the paranasal sinus [13]. The authors of that report used a vascularized nasoseptal flap via a combined transcranial and EEA approach in an effort to minimize the risk of a postoperative cerebrospinal fluid leak [13]. However, in our case, we chose a staged procedure in view of the patient’s preoperative hemiplegia and underlying comorbidities. The sphenoid sinus tumor in this patient had expanded extensively into the sphenoidal lateral fossa, so we planned a strategy whereby we could perform the least aggressive and most effective endoscopic endonasal surgery consisting of endoscopic modified medial maxillectomy via a transpterygoidal, pterygopalatine fossa, and sphenoid sinus lateral fossa approach. Using this combined approach, we successfully removed the tumor in the sphenoid without any leak of cerebrospinal fluid.

Pathological examination identified the resected tumor to be a World Health Organization grade I transitional meningioma, which is reported to be less likely to recur than tumors with a poorer grade [1]. The MIB-1 index, which characterizes proliferation, was less than 1% at the time of the first operation, 5% at the second, and 15% at the third. When the MIB-1 index demonstrates a tendency to increase, careful observation is required thereafter [14].

Postoperative neurological examination revealed no neurological deficits, with normal ocular movement and facial sensation. We consider the favorable outcome in this patient to have been achieved by separate staged surgery for the intracranial tumor and the tumor that developed in the paranasal sinus. However, it would be important to make every effort to ensure that the patient is not left with sequelae when this surgical strategy is used.

## 4 Conclusion

We found that a large sphenoid ridge meningioma extending into the sphenoid sinus could be removed successfully in a minimally invasive manner via a transsphenoidal and transpterygoid approach. This approach was useful for ensuring sufficient visualization of the surgical field for removal of a sphenoid sinus tumor that had expanded extensively into the sphenoidal lateral fossa.

## Declaration of Competing Interest

There are no conflicts of interest to report.

## Sources of funding

This research did not receive any specific grant from funding agencies in the public, commercial, or not-for-profit sectors.

## Ethical approval

This report describes established surgical procedures and therefore does not require ethical approval at our institution (Kyorin University).

## Consent

Our manuscript included a statement at the end of the manuscript, as follows: "Written informed consent was obtained from the patient for publication of this case report and accompanying images. A copy of the written consent is available for review by the Editor-in-Chief of this journal on request”.

## Author’s contribution

Hidenori Yokoi: writing - preparation of the original draft, operating surgeon, investigation, methodology.

Satoru Kodama: operating surgeon.

Keisuke Maruyama: operating surgeon.

Masachika Fujiwara: pathologic analysis.

Yoshiaki Shiokawa: supervision.

Koichiro Saito: supervision.

## Registration of research studies

Our manuscript is a case report and therefore, does not require registration.

## Guarantor

Dr. Hidenori Yokoi is the guarantor for this study.

## Provenance and peer review

Not commissioned, externally peer-reviewed.
